# IFN-α potentiates the direct and immune-mediated antitumor effects of epigenetic drugs on both metastatic and stem cells of colorectal cancer

**DOI:** 10.18632/oncotarget.8379

**Published:** 2016-03-25

**Authors:** Maria Buoncervello, Giulia Romagnoli, Mariachiara Buccarelli, Alessandra Fragale, Elena Toschi, Stefania Parlato, Donatella Lucchetti, Daniele Macchia, Massimo Spada, Irene Canini, Massimo Sanchez, Mario Falchi, Martina Musella, Mauro Biffoni, Filippo Belardelli, Imerio Capone, Alessandro Sgambato, Lucia Ricci Vitiani, Lucia Gabriele

**Affiliations:** ^1^ Department of Hematology, Oncology and Molecular Medicine, Istituto Superiore di Sanità, Rome, Italy; ^2^ Istituto di Patologia Generale, Università Cattolica del Sacro Cuore, Rome, Italy; ^3^ Department of Cell Biology and Neurosciences, Istituto Superiore di Sanità, Rome, Italy; ^4^ National AIDS Center, Istituto Superiore di Sanità, Rome, Italy

**Keywords:** colorectal cancer, cancer stem cell, interferon, epigenetics, immunogenic cell death

## Abstract

Epigenetic alterations, including dysregulated DNA methylation and histone modifications, govern the progression of colorectal cancer (CRC). Cancer cells exploit epigenetic regulation to control cellular pathways, including apoptotic and metastatic signals. Since aberrations in epigenome can be pharmacologically reversed by DNA methyltransferase and histone deacetylase inhibitors, epigenetics in combination with standard agents are currently envisaged as a new therapeutic frontier in cancer, expected to overcome drug resistance associated with current treatments. In this study, we challenged this idea and demonstrated that the combination of azacitidine and romidepsin with IFN-α owns a high therapeutic potential, targeting the most aggressive cellular components of CRC, such as metastatic cells and cancer stem cells (CSCs), via tight control of key survival and death pathways. Moreover, the antitumor efficacy of this novel pharmacological approach is associated with induction of signals of immunogenic cell death. Of note, a previously undisclosed key role of IFN-α in inducing both antiproliferative and pro-apoptotic effects on CSCs of CRC was also found. Overall, these findings open a new frontier on the suitability of IFN-α in association with epigenetics as a novel and promising therapeutic approach for CRC management.

## INTRODUCTION

Colorectal cancer (CRC) develops upon a multistep process in which genetic mutations and epigenetic alterations drive tumor initiation, progression and metastatic growth [1-3]. Typically, KRAS mutations are key elements in determining CRC aggressiveness and resistance to therapy, since they activate cell proliferation, differentiation, survival, migration and apoptosis [4]. Another key factor contributing to CRC aggressiveness is the elevated degree of cell heterogeneity due to both poorly differentiated metastatic cells, capable of migrating and invading basal lamina, and cancer stem cells (CSCs), a highly tumorigenic population with elevated self-renewing capability [5, 6]. Of interest, these cell populations share molecular characteristics, drive metastasis formation and confer resistance to conventional drug therapies [7]. Recently, epigenetic alterations have been discovered as key factors in CRC pathogenesis [8-11]. Accumulating evidence indicates that abnormal changes in DNA methylation and histone modifications may act in concert in regulating expression of genes that drive the tumorigenic process [12]. Since both histone modifications and DNA methylation are potentially reversible by pharmacological treatments, they represent attractive targets for therapeutic strategies. On this basis, the DNA methyltransferase inhibitor (DNMTi) azacitidine and the histone deacetylase inhibitor (HDACi) romidepsin have been approved by FDA for treating patients with myelodysplastic syndrome and cutaneous T-cell lymphoma, respectively [13], and are currently under evaluation for solid tumors, such as glioblastoma, renal and lung cancer [14, 15]. Because of the limited results as monotherapies, a more rational use of epigenetic drugs foresees the combination with other therapies, also in the perspective of lowering doses to override high dose-associated cytotoxic and off-target effects [16-18]. Given the potential of epigenetic drugs to stimulate the immune system, their combination with immunomodulators may be regarded as a promising new frontier [19-21]. In this context, IFN-α2b (IFN-α) represents one of the most suitable candidate for such association, due to its potent direct and immune-mediated antitumor activities [22]. Noteworthy, a tight crosstalk between IFN-α and epigenetic signatures as well as the dysfunctional induction of IFN-α response in CSCs have been recently reported to associate with tumor progression [23-25].

Immunogenic cell death (ICD), a type of apoptosis characterized by high potential of stimulating immune cells, has emerged as one of the most powerful cellular processes capable to strengthen the anti-tumor specific immune response [26]. Only some chemotherapeutics or radiation are endowed with the ability to trigger ICD [27]. Recently, type I IFN (IFN-I) signaling has emerged as mandatory to achieve ICD-mediated antitumor response to anthracycline [28]. Hence, IFN-α owns the potential to cooperate with other therapeutic strategies, by supporting killing of cancer cells while generating immunogenic signals [29].

Herein, we investigated the capability of IFN-α to cooperate with azacitidine and romidepsin in hampering the aggressiveness of both metastatic cells and CSCs in CRC. We found that IFN-α potentiates the antiproliferative and pro-apoptotic properties of azacitidine and romidepsin *in vitro* and it is essential for triggering cell death with immunogenic features, ultimately improving dendritic cell (DC) phagocytosis of drug-treated cancer cells. Lastly, IFN-α cooperates with both drugs to inhibit tumor cell growth *in vivo*.

## RESULTS

### IFN-α enhances the growth inhibitory activity of azacitidine and romidepsin on both metastatic cells and CSCs of CRC

**Figure 1:**
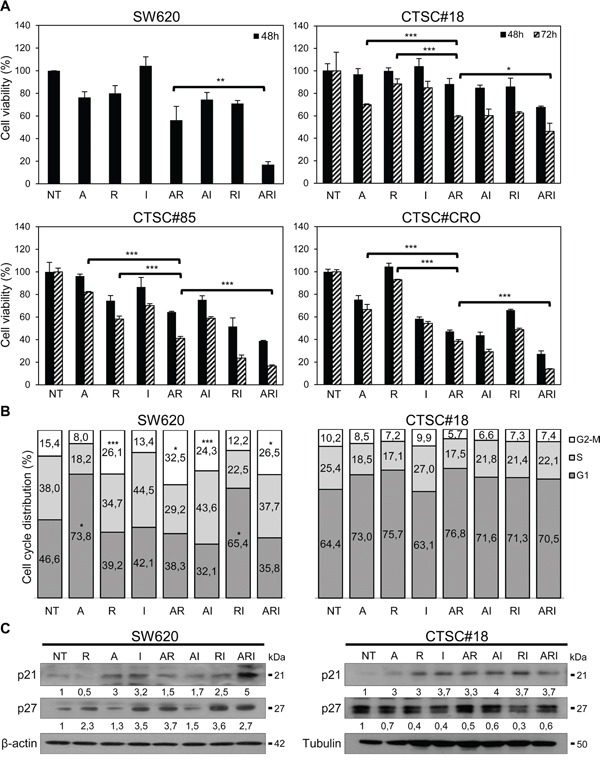
IFN-α potentiates the antiproliferative effects exerted by azacitidine and romidepsin on both metastatic cells and CSCs of CRC **A.** The antiproliferative effects of drugs on SW620, CTSC#18, CTSC#85 and CTSC#CRO cells were evaluated by MTS assays. Cells were treated with azacitidine (A), romidepsin (R) and IFN-α (I), as single agents or in combination [A: 10 μM (SW620), 1 μM (CTSC#18, CTSC#85 and CTSC#CRO); R: 2 nM (SW620), 0.6 nM (CTSC#18, CTSC#85 and CTSC#CRO); I: 10000 IU/ml]. Viability of cells was assayed upon drug treatments for 48 h in SW620 and for 48 and 72 h in CTSC#18, CTSC#85 and CTSC#CRO. Experiments were performed in triplicate and drug-treated values were normalized to untreated cells (NT), at each time point. Each value represents the mean ± S.D. of three independent experiments. **B.** Drugs effects on cell cycle of SW620 and CTSC#18 cells were evaluated by DNA flow cytometric analysis. Exponentially growing cells were analyzed after 48 h of treatment with A, R and I, alone or in combination. Data represent the mean percentage of cells in each phase of the cell cycle of four independent experiments. **C.** Western blotting analysis of the cell cycle inhibitors p21^Waf1/Cip1^ and p27^Kip1^, following single and combined treatments of SW620 for 48 h and CTSC#18 for 72 h. β-actin (SW620) or tubulin (CTSC#18) were used as loading controls. Intensities of bands were measured and values, normalized to housekeeping proteins, are expressed as arbitrary unit (AU) at the bottom of each panel. One representative experiment of three is shown. *P ≤ 0.05; **P ≤ 0.01; ***P ≤ 0.001.

To investigate whether IFN-α could cooperate with azacitidine and romidepsin in hampering the metastatic potential of KRAS-mutated CRC cells, we first identified the effective combination dose with the minimum potential toxicity. To this end, we measured cell viability by MTS assays and defined the lowest dose of each agent, ranging between IC20 and IC10, effective on both KRAS-mutated metastatic SW620 cells and CTSC#18, a CSC line derived from a CRC patient [30] (Table 1). SW620 and CTSC#18 cells resulted differently sensitive to azacitidine and romidepsin, being the former early responders at higher drug doses and the latter susceptible to lower drug doses at later time points (Supplementary Figure 1). Comparable and even lower ranges of drug-sensitivity were found for the KRAS-mutated poorly invasive SW480 cells and for three KRAS-wild-type CSC lines (CTSC#1.2, CTSC#85, CTSC#CRO) (Supplementary Table 1). Together, these results indicate that KRAS mutation confers a relative resistance to all tested agents as single treatments. Therefore, we tested the effects of combined azacitidine, romidepsin and IFN-α (hereafter: ARI) in SW620 and CTSC#18 cells, owing simultaneously high metastatic potential and elevated resistance to the antiproliferative effects of the single agents. We found that while azacitidine and romidepsin combined treatment (AR), with respect to the single agents, slightly inhibited SW620 and CTSC#18 proliferation, the addition of IFN-α to the epigenetic agents drastically enhanced these antitumor effects (Figure 1A). Of note, the KRAS-wild-type CTSC#85 and CTSC#CRO CSC lines exhibited even a higher sensitivity to ARI or IFN-α in combination with romidepsin (RI) (Figure 1A). These data were further confirmed in SW480 and CTSC#1.2 cells (Supplementary Figure 2). Next, we investigated the mechanisms underlying the antitumor cooperation between IFN-α and epigenetic drugs. We found that, while azacitidine determined a significant arrest of SW620 cells in G2/M and romidepsin promoted accumulation of cells in the G1 phase, IFN-α was unable either to influence cell cycle progression, as single agent, or to interfere with the predominant activity of romidepsin on azacitidine (Figure 1B). Nevertheless, IFN-α, alone or in RI and ARI combinations, stimulated induction of the cell cycle inhibitors p21^Waf1/Cip1^ and p27^Kip1^ (Figure 1C). Of interest, CTSC#18 cells did not show any cell cycle arrest and exhibited broad up-modulation of only p21^Waf1/Cip1^ upon any treatment (Figure 1B and 1C).

**Table 1:** IC10-IC20 drug values in SW620 and CTSC#18 cells at 48 h and 72 h, respectively

Treatments	SW620	CTSC#18
Azacitidine	10 μM	1 μM
Romidepsin	2 nM	0.6 nM
IFN-α	10000 IU/ml	10000 IU/ml

Data represent the mean of at least 3 independent experiments.

### ARI combined treatment strongly inhibits invasive signaling pathways in both metastatic cells and CSCs of CRC

**Figure 2:**
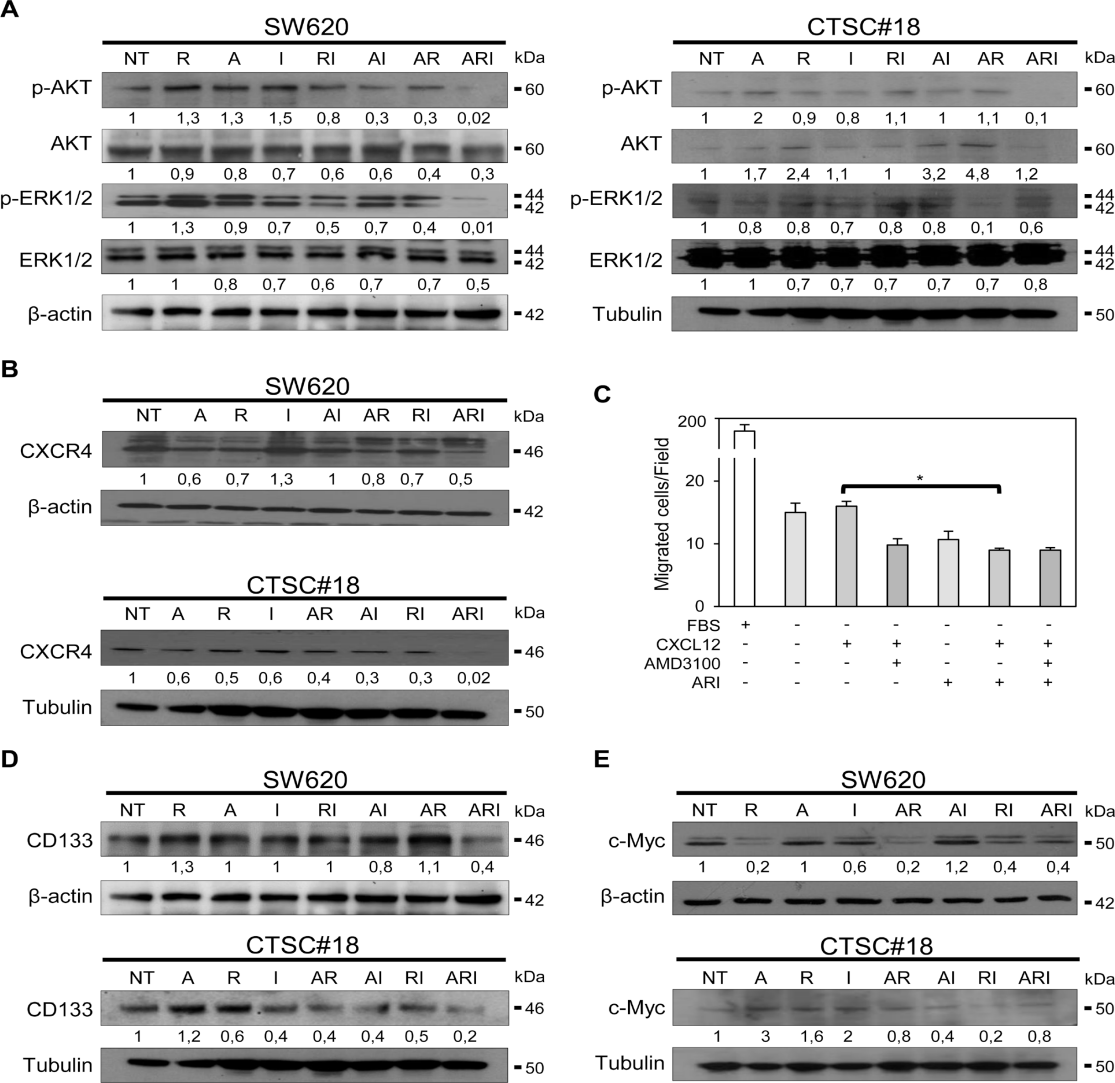
Azacitidine, romidepsin and IFN-α cooperate in shutting-down the main metastatic signaling pathways in both metastatic cells and CSCs of CRC **A.** p-AKT, AKT, p-ERK1/2, ERK1/2 protein expression was detected by western blotting analysis of cell lysates of SW620 and CTSC#18 cells, NT or drug-treated for 24 h and 72 h, respectively. **B.** CXCR4 protein level was evaluated by western blotting analysis of lysates of SW620 and CTSC#18 cells, NT and drug-treated for 48 h and 72 h, respectively. **C.** The migration rate of SW620 cells, NT or ARI-treated for 72 h, was tested towards an exogenous gradient of CXCL12 (200 ng/ml), under serum-free conditions in the presence or absence of AMD3100 (5 μM). Data are expressed as mean number of migrated cells. **D, E.** CD133 and c-Myc protein expression assessed by western blotting analysis of lysates of SW620 and CTSC#18 cells, NT and drug-treated for 24 h and 72 h, respectively. In all experiments, β-actin or tubulin were included as internal control for SW620 and CTSC#18, respectively. Intensities of bands were measured and values, normalized to housekeeping proteins, are expressed as AU at the bottom of each panel. One representative experiment of three is shown. *P ≤ 0.05.

We investigated the effects of ARI treatment on the phosphatidylinositol 3-kinase (PI3K)/AKT-ERK1/2 survival pathway, pivotal for maintaining CRC cell proliferation and invasion [31]. As shown in Figure 2A, ARI combination decreased the levels of p-AKT/AKT and p-ERK1/2/ERK1/2 in both SW620 and CTSC#18 cells. We also found that this triple drug combination significantly decreased the expression of CXCR4, another signal known to govern the metastatic phenotype of CRC cells, partially via AKT-ERK1/2 pathway [32, 33] (Figure 2B). Accordingly, ARI-treated SW620 cells, with respect to untreated cells, exhibited a clear impaired ability to migrate, even in presence of CXCL12 (Figure 2C). As CD133^+^CXCR4^+^ cells have been associated with poor 2-year survival of CRC patients [34], we also evaluated the modulation of CD133 and found its strong reduction in both SW620 and CTSC#18 cells 24 and 72 h after treatment, respectively (Figure 2D). Down-modulation of CD133 and CXCR4 surface expression in both types of CRC cells upon ARI treatment was also confirmed by flow cytometry (Supplementary Figure 3). Moreover, ARI was the only treatment able to counteract the propensity of IFN-α and azacitidine to slightly increase the expression of c-Myc, a pivotal epigenetic-regulated transcription factor whose expression is directly correlated with the metastatic phenotype of CRC cells [35] (Figure 2E).

### ARI treatment induces high rate of apoptosis

**Figure 3:**
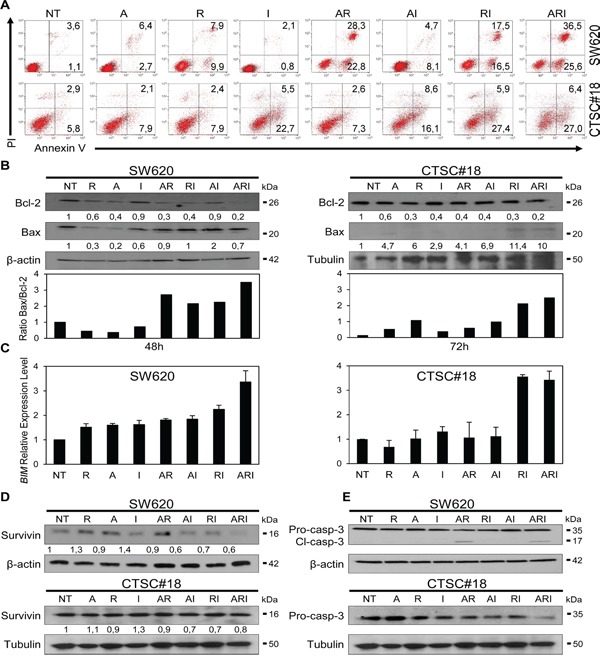
IFN-α potentiates the pro-apoptotic effects of azacitidine and romidepsin in metastatic cells and CSCs of CRC **A.** The apoptotic rate of NT and drug-treated SW620 and CTSC#18 cells was established by FACS analysis. Cells were treated with drugs for 48 h and then stained for apoptosis detection. In the flow plots, early apoptotic cells (Annexin V^+^/PI^−^), late apoptotic cells (Annexin V^+^/PI^+^), necrotic cells (Annexin V^−^/PI^+^) and vital cells (Annexin V^−^/PI^−^) can be distinguished. One representative experiment out of five independent experiments is shown. **B.** Bcl-2 and Bax protein expression evaluated by western blotting analysis of lysates of SW620 and CTSC#18 cells, NT or cells treated with A, R and I, alone or in combination, for 48 h and 72 h, respectively. One representative experiment out of three is shown. Bax/Bcl-2 expression ratio was evaluated on the normalized values and shown in the bottom panel. **C.** qRT-PCR analysis of *BIM* transcripts in SW620 and CTSC#18 cells. Amounts of mRNA transcripts were expressed in relative copy numbers normalized to the housekeeping gene GAPDH (comparative Ct method, ΔCt). Experiments were performed in triplicate, and each value, normalized to GAPDH, represents mean ± S.D. of three independent experiments. **D, E.** Expression of Survivin and pro–caspase/cleaved-Caspase-3 was evaluated by western blotting analysis of SW620 and CTSC#18 cells, NT and drug-treated for 48 h and 72 h, respectively. Intensities of bands were measured and values normalized to β-actin or tubulin are expressed as AU at the bottom of each panel. One experiment out of three is shown.

Since one of the major objectives of therapeutic treatments is to overcome the resistance of cancer to cell death [36, 37], we investigated whether IFN-α was capable to potentiate any pro-apoptotic effect eventually exerted by the epigenetic drugs. After 48 h exposure, IFN-α was found to cooperate with both AR and romidepsin alone in enhancing significantly the apoptotic rate of SW620 cells (Figure 3A). Noteworthy, in CTSC#18 cells IFN-α exhibited a strong capability to stimulate apoptosis already as mono-treatment and enhanced the pro-apoptotic properties mainly of romidepsin in both ARI an RI combinations, disclosing the propensity of these cells, usually highly apoptotic-resistant, to undergo apoptosis upon exposure to these drugs (Figure 3A). Accordingly, we observed the modulation of anti- and pro-apoptotic proteins following ARI treatment. In particular, the anti-apoptotic Bcl-2 was decreased in both SW620 and CTSC#18 cells, while the pro-apoptotic Bax remained relatively stable in SW620 and was increased in CTSC#18 cells, resulting in both cases in a significant enhancement of the Bax/Bcl-2 ratio (Figure 3B). We also found a strong up-modulation of *BIM*, another member of the BCL-2 family, whose loss has been reported to be associated with resistance to HDACi therapy [38] (Figure 3C). Interestingly, in CTSC#18 cells *BIM* was increased as well upon RI treatment. Moreover, Survivin, another apoptotic inhibitor protein correlated to cell death evasion occurring upon most of the current therapies in CRC [39, 40], was significantly decreased in SW620 cells upon ARI treatment, while a light decrease of this marker was observed in CTSC#18 cells (Figure 3D). Lastly, activation of Caspase-3 was observed in both cell types, although in SW620 the AR combination was sufficient to induce this event (Figure 3E). The involvement of Caspase-3 in ARI-induced apoptosis of SW620 cells was further confirmed by the inhibition, at least in part, of this process with the specific inhibitor z-DEVD-FMK (Supplementary Figure 4). Overall, our results suggest that IFN-α owns the potential to cooperate with epigenetic drugs in inducing apoptosis by modulation of key apoptotic molecules.

### ARI treatment drives ICD, increases the rate of DC phagocytosis of drug-treated CRC cells and inhibits *in vivo* tumor growth

**Figure 4:**
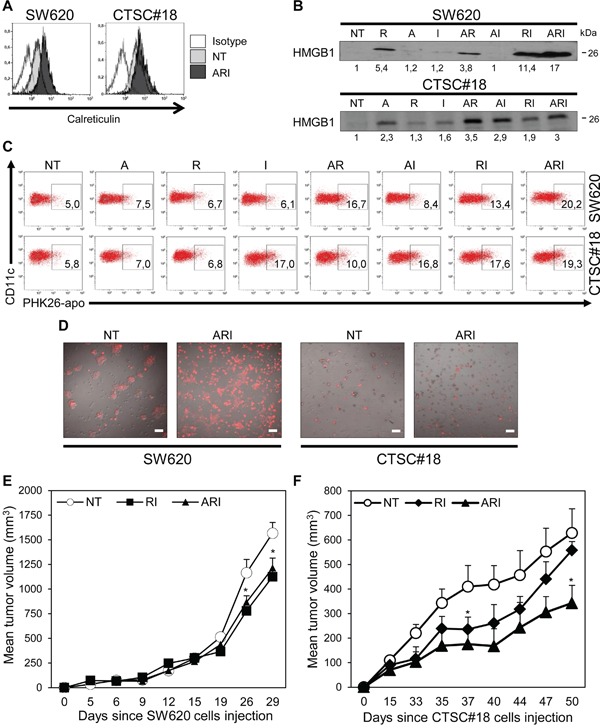
Azacitidine, romidepsin and IFN-α combined treatment induces ICD signals, enhances DC uptake of drug-treated CRC cells and inhibits *in vivo* growth of CSCs **A.** CRT expression was evaluated as mean fluorescence intensity of live cells (PI^−^ gate), NT or ARI-treated for 72 h, by FACS analysis. Data are representative of three independent experiments. **B.** HMGB1 extracellular release was assayed by western blotting analysis of SW620 and CTSC#18 supernatants cells, NT or drug-treated for 48 h and 96 h, respectively. One representative experiment out of three is shown. **C.** The phagocytic capacity of IFN-α-DCs was evaluated after 4 h co-cultures with PHK26-labeled SW620 or CTSC#18 cells, NT and drug-treated for 48 h. After washes and DC staining with anti-CD11c Ab, phagocytosis was evaluated by flow cytometry. Data are representative of three independent experiments. **D.** CLSM analysis of IFN-α-DCs co-cultured with PHK26-labeled SW620 or CTSC#18 cells, NT and ARI-treated for 48 h; cells were fixed and images were observed through a 20X objective lens. Data are representative of three independent experiments. Scale bar, 30 μm. **E, F.** *In vivo* CRC cells growth is inhibited by ARI treatment. NOD-SCID mice were injected *s.c.* with 2×10^6^ SW620 and 1×10^6^ CTSC#18 cells and tumor size was measured over time. After 6 and 14 days respectively, mice were treated *i.p.* with A (0.25 mg/kg), R (0.32 mg/kg) and I (300000 IU/kg) in ARI and RI combination twice a week for three weeks. Data represent the mean tumor volume ± S.E.M. One representative experiment out of three is shown. *P ≤ 0.05, with respect to NT cells.

Because it has been reported that ICD is a crucial component of some antineoplastic treatments and that IFN-I are implicated in this process [29, 41], we next evaluated the capability of IFN-α to cooperate with epigenetic drugs in inducing this type of cellular demise. Thus, we assessed the translocation of Calreticulin (CRT) from the lumen of ER vesicles to the surface of tumor cells and the release of HMGB1, key hallmarks of ICD [26, 41, 42]. The flow cytometry analysis revealed that ARI combination, with respect to single and double treatments, was able to stimulate CRT membrane translocation in both SW620 and CTSC#18, 72 h after exposure to drugs (Figure 4A and Supplementary Figure 5). Of interest, IFN-α, when in ARI or RI combinations, significantly increased the capability of romidepsin to induce HMGB1 release in SW620 cells (Figure 4B). On the other hand, in CTSC#18 cells, IFN-α was able to induce this signal already as single agent and to potentiate it in AI and RI combinations, whereas no additive effects were observed upon ARI triple treatment (Figure 4B). Altogether, these results suggest that, although ARI treatment induces stronger immunogenic signals in metastatic cells than CSCs, these latter display a remarkable susceptibility already to IFN-α as mono-treatment. Next, we evaluated whether the immunogenic signals induced by the combination of IFN-α with epigenetic drugs could be converted into enhanced DC phagocytosis of drug-treated CRC cells. We found that human DCs differentiated from peripheral monocytes in the presence of IFN-α (IFN-α-DCs), endowed with a high capability in inducing antitumor CD8^+^ T cell response [43], were able to phagocyte, at very high rates, both ARI-treated SW620 and CTSC#18 (Figure 4C). Noteworthy, CTSC#18 cells exposed to IFN-α as single agent were already efficiently taken-up by IFN-α-DCs (Figure 4C). The strong propensity of IFN-α-DCs to phagocytose both ARI-treated metastatic cells and CSCs was further confirmed by confocal laser scanning microscopy (CLSM) analysis (Figure 4D). Finally, to better investigate the antitumor effects of combined epigenetic drugs and IFN-α we performed *in vivo* experiments into SW620- and CTSC#18-transplanted NOD-SCID mice (Supplementary Figure 6). We found that ARI combination was effective in causing a significant delay in the growth of both types of tumors, although RI exhibited equally antitumor activity (Figure 4E and 4F). This finding validates the remarkable direct antitumor capabilities of ARI and RI combinations.

## DISCUSSION

This study provides the first evidence that IFN-α cooperates with epigenetic drugs in inducing direct and immune-mediated antitumor effects on both metastatic cells and CSCs of CRC. In particular, we report that IFN-α in combination with epigenetic drugs inhibits the proliferation and the metastatic behavior of KRAS-mutated highly metastatic SW620 cells and patient-derived self-renewing CTSC#18 CSCs, by two main mechanisms: (i) shutting down metastatic cellular pathways, including CXCR4, ERK1/2 and AKT signals; (ii) inducing apoptosis with ICD features able to deliver signals to DCs that, in turn, increase their capability to phagocytose drug-treated cancer cells. Noteworthy, the other most striking and innovative observation of this study is the capability of IFN-α as mono-treatment to exert remarkable antiproliferative and pro-apoptotic effects on CSCs.

The vast majority of diagnosed CRC is represented by non-hereditary “sporadic cancers”, characterized by a progressive accumulation of multiple genetic and epigenetic alterations within tumor cells [44]. Among them, KRAS mutations are key activators of cell proliferation and metastasis driving CRC progression [45]. Epigenetic mechanisms are linked to isoform-specific KRAS proteins, that drive different cancer programs by modulation of diverse downstream signaling pathways [46]. Of interest, epigenetic alterations may act in concert to regulate expression of genes implicated in cell cycle, apoptosis, invasion and metastasis of almost all types of CRC [47, 48]. In particular, deregulation of enzymes mediating epigenetic alterations has been associated with progression as well as clinical outcome of CRC [49, 50]. Likewise, high expression of HDACs is associated with reduced survival of CRC patients [51]. Because epigenetic alterations are reversible by blocking the activity of epigenetic enzymes, both DNMTi and HDACi, such as azacitidine and romidepsin, have been exploited in diverse hematological malignancies and are currently being tested in patients with solid cancers, including CRC [52]. However, data collected from initial trials suggest that azacitidine, utilized at maximally tolerated doses, associates with extensive toxicity and minimal efficacy, whereas it is effective in inhibiting tumor-specific DNA hypermethylation at lower doses [15]. Similarly, while romidepsin as monotherapy exerts minimal anticancer activity with important side-effects, its use at lower doses has showed great potential in modulating gene expression [53]. Recently, combination of DNMTi and HDACi with immunotherapy has been regarded as an innovative therapeutic frontline [20]. In the present study, we challenged this idea and found that the antitumor effects of azacitidine and romidepsin, at low doses, are strongly potentiated by IFN-α. In particular, ARI combination determines in both metastatic cells and CSCs strong inhibition of CXCR4, AKT and ERK1/2 pathways, all invasive signals associated with CRC progression, advanced tumor stage, lymphatic recurrence and poor prognosis [54, 55]. Moreover, ARI down-modulates both CD133, a well-known CSC marker whose association with CXCR4 defines a subset of highly metastatic CSCs [34], and c-Myc, one of the most critical transcription factor implicated in CRC transformation [35].

Of note, the modulation of these signals are associated with cell cycle arrest and up-modulation of p21^Waf1/Cip1^ and p27^Kip1^ only in SW620 cells, whereas a moderate up-modulation of p21^Waf1/Cip1^ is observed in CTSC#18 cells. These findings suggest that the growth inhibitory effect exerted by ARI on metastatic cells and CSCs may occur through different signaling pathways, as observed in other cancer cells upon exposure to azacitidine or decitabine in combination with FAS-ligand [56].

In this study, we also report for the first time the cooperation between IFN-α and epigenetic agents in promoting cell death of highly invasive CRC cells, by the simultaneous control of cellular pathways conveying apoptosis resistance [39]. However, while ARI is the most effective treatment in inducing high rate of apoptosis in metastatic SW620 cells, both ARI and RI combinations stimulate high levels of apoptotic cell death in CTSC#18 CSCs. Importantly, in both cell models apoptosis occurs with significant down-modulation of a number of typical anti-apoptotic regulators, such as Bcl-2, Bim and Survivin, and increased levels of the pro-apoptotic Bax, along with activation of Caspase-3. In this regard, because the activation of CXCR4, ERK1/2 and AKT signals has been associated with apoptosis resistance through Survivin up-modulation [57], ARI treatment appears to be a particularly suitable pharmacological option to break this survival axis. Moreover, the antitumor potential of this drug combination is strengthened by its ability to interrupt apoptosis resistance associated to c-Myc expression [58]. Overall, ARI combination appears as a therapeutic strategy capable of modulating the most common cellular signals responsible for CRC therapeutic resistance, likely by re-establishing a correct dialogue between epigenetic modifications and cellular transcriptional activity.

Recently, ICD has emerged as a peculiar form of apoptosis able to stimulate an efficacious adaptive immune response against cancer cell-associated antigens [29], and IFN-α has been reported among the few agents that own the property to induce it [27]. Here, we report that IFN-α is crucial in sustaining epigenetic-driven induction of ICD, in both metastatic cells and CSCs. In particular, IFN-α exhibits the capability to strengthen significantly the basal propensity of romidepsin and azacitidine to induce HMGB1 release, in SW620 and CSCs respectively, and to promote late CRT membrane exposure when it is in combination with both epigenetics. Of interest, the belated CRT surface exposure may represent an immunologic hallmark specifically induced by low doses of combined drugs, as reported for sublethal chemotherapeutic treatment [59]. Noteworthy, the need of ARI combination for stimulating ICD signals confirms recent findings pointing out the requirement of the immune system for the therapeutic efficacy of epigenetic agents [60].

The translation of ICD into a therapeutic advantage relies on the capacity of DCs, recruited to sites of ongoing cancer cell death, to take up apoptotic bodies and prime an adaptive immune response [61]. In this regard, over the past years IFN-α has emerged as a key inducer of differentiation and activation of DCs and, accordingly, IFN-α-DCs are endowed with a marked phagocytic activity and a special aptitude in inducing CD8^+^ T-cell response [43]. In this study, we report that the phagocytosis rate of IFN-α-DCs significantly increases only in the presence of ARI-treated metastatic cells or CSCs. Of note, IFN-α-DCs are also able to take up with great efficacy CSCs exposed to IFN-α alone, fully in agreement with the capacity of this cytokine to stimulate high HMGB1 release by CSCs. Overall, from CSCs point of view, our data provide for the first time the evidence that these cells are highly sensitive to the antitumor effects of exogenous IFN-α, alone or in combination with epigenetic drugs, primarily romidepsin. This cooperation is further confirmed by the significant inhibition of tumor growth *in vivo* by ARI and, to a lesser extent, RI treatment.

Hence, we envisage that the strength of ARI combination relies on re-establishing a normal “epigenetic landscape” by the simultaneous use of azacitidine and romidepsin, which leads to unlock specific genes allowing IFN-α-induced transcriptional activity, with the final result to control most of the cellular signals deregulated during CRC development and progression. Importantly, these signals converge with the induction of ICD, bridging direct and immune-mediated antitumor effects. These findings are also supported by two recent reports highlighting the key role of IFN signaling in the antiproliferative response to DNA demethylation in both ovarian and CRC cells [62, 63]. Noteworthy, although IFN-α and epigenetic drugs in combination exert significant antitumor activity in both metastatic cells and CSCs, the traits of this response own some differences in quality and timing between the two types of CRC cells, suggesting the potential to activate selective signals in different components of the tumor mass. While future work will be necessary to clarify the bases of these events, our data clearly support the complementarity between epigenetic- and immune-targeting agents in inducing concomitantly direct and immune-mediated antitumor effects and open a new frontier for the management of CRC as well as other solid tumors.

## MATERIALS AND METHODS

### Cell cultures

Low passage-number cell lines SW480 and SW620, obtained from American Type Culture Collection, were maintained in RPMI-1640 medium (Lonza, Verviers, Belgium) supplemented with 10% FBS (EuroClone, West York, United Kingdom) in the presence of penicillin and streptomycin. Cells were seeded at 3.5×10^4^/cm^2^ and the day after treated with 10 μM azacitidine, 2 nM romidepsin and 10^4^ IU/ml IFN-α alone or in combination. CSC lines of CRC were generated by Dr Lucia Ricci-Vitiani as previously described [30] and cultured in a serum-free medium supplemented with 20 ng/ml EGF and 10 ng/ml FGF-2 (PeproTech, Rocky Hill, NY). CSC lines were validated by Short Tandem Repeat DNA fingerprinting. For all CSC lines, profiles were compared against publically available databases to confirm authenticity (Biological Bank and Cell Factory, National Institute for Cancer Research, IST, Genova, Italy). Both types of cells were either collected for quantitative reverse transcriptase PCR (qRT-PCR), immunofluorescence staining and immunoblotting at 24, 48, or 72 h upon epigenetic and IFN-α treatments.

### Drug sources

Azacitidine was purchased from Sigma-Aldrich (St Louis, MO, USA), romidepsin from Selleckchem (Houston, TX, USA) and IFN-α (Intron A) from Merck Sharp and Dohme (Kenilworth, NJ, USA).

### Cell viability assay

CellTiter 96 AQueous One Solution Cell Proliferation Assay (Promega, Madison, WI) was used to determine cytotoxicity effects after treatment with drugs. Absorbance at 450 nm was determined on Opsys MR spectrophotometer (DYNEX Technologies, Denkendorf, Germany), using Windows Revelation QuickLink Software. SW480 and SW620 cell lines were plated at a density of 4×10^3^ and 4.5×10^3^ cell/well, respectively, in 96-well plates, and then treated with various concentrations of azacitidine, romidepsin and IFN-α for 48 and 72 h. At the end of treatments, 20 μl of 3-(4,5-dimethylthiazol-2-yl)-5-(3-carboxymethoxyphenyl)-2-(4-sulfophenyl)-2H-tetrazolium (MTS) reagent were added to each well. The plates were then incubated for 2 h at 37°C in the dark. Each experimental condition was performed in triplicate and repeated at least twice. All values were normalized with respect to the viability of untreated cells. For cytotoxicity analysis, CSCs were enzymatically dissociated using TrypLE™ Express Enzyme 1X (Gibco/ThermoFisher Scientific, Waltham, MA) and plated at 3×10^4^ cells/ml density, in triplicate, in 96-well plates, treated with various concentrations of azacitidine, romidepsin and IFN-α alone or in combination for 48 and 72 h. ATP levels were measured as a marker of cell viability using the CellTiter-Glo™ (Promega) in accordance with the manufacturer's instructions.

### RNA preparation and qRT-PCR

Total RNA was isolated from cells with TRIsureTM (Bioline, London, UK), according to the manufacturer's instructions, quantified using NanoDrop ND-1000 (Thermo Scientific, USA) and converted to cDNA by random primers and ThermoScript reverse transcriptase (Invitrogen, Carlsbad, CA). qRT-PCR analysis was performed using the Applied Biosystems 7500HT Real-Time PCR System. *BIM* expression was analyzed by using the primer sequences: 5′-TGGCAAAGCAACCTTCTGATG-3′ and 5′-GCAGGCTGCAATTGTCTACCT-3′. PCR reactions were prepared by using SensiMix SYBR Kit (Bioline). Amounts of mRNA transcripts were expressed as absolute copy numbers normalized to the housekeeping gene GAPDH (ΔCt method).

### Immunoblotting

Protein expression was assessed by lysing cells with a high salt lysis buffer containing 500 nM Tris-HCl (pH 7.4), 300 nM NaCl, 1% NP40, 5 mM EDTA, and a protease inhibitor mixture (Roche Diagnostics, Basel, Switzerland). Equal amounts of total proteins were boiled in sample buffer and separated by sodium dodecyl sulfate–polyacrylamide gel electrophoresis (SDS-PAGE). To detect specific protein expression, the following antibodies (Abs) were used: c-Myc, Survivin, Caspase-3, p27, Tubulin (Santa Cruz Biotech, CA), CXCR4, HMGB1 (Abcam, Cambridge, United Kingdom), AKT, p-AKT, ERK1/2, p-ERK1/2, CD133, Bax, p21 (Cell Signaling Technology, Danvers, MA), Bcl-2 (Dako, Glostrup, Denmark), β-actin (Sigma-Aldrich). Immunoreactive bands were visualized by using HRP-conjugated secondary Ab and the ECL system (Amersham Biosciences, Buckingham, United Kingdom). HMGB1 expression was analyzed in cell supernatants. Briefly, equal amounts of each sample normalized by cell number were separated by SDS-PAGE, and immunoblot analysis was performed with a rabbit polyclonal Ab to HMGB1, as described [18]. The optical density of the bands was measured by using the National Institutes of Health Image J software (rsb.info.nih.gov/ij).

### Cell migration assay

SW620 cell line was treated for 72 h with ARI treatment. Cells were then harvested with trypsin, washed with chilled Phosphate Buffered Saline (PBS) pH 7.4 and resuspended in serum-free medium containing 0.5% bovine serum albumin (migration medium), with or without AMD3100 (5 μM, Sigma-Aldrich), and seeded in duplicate (1×10^5^ cells/chamber) in the upper compartment of Boyden chambers. The lower compartment was filled with migration medium containing or not 200 ng/ml human recombinant CXCL12 (R&D Systems, Inc). The upper and lower compartments were separated by 8 μm pore size polycarbonate filters (Nucleopore, Whatman, Clifton, NJ) coated with 20 μg/ml Collagen type IV (Sigma-Aldrich) and 10 μg/ml fibronectin (Sigma-Aldrich). After 2 h of migration, non-invaded cells presented on the upper surface of the filters were mechanically removed, whereas cells that had migrated to the lower surface were fixed in 4% paraformaldehyde (EuroClone) and stained with toluidine blue (Sigma-Aldrich). The numbers of invaded cells were counted in ten different fields (magnification 40X).

### Cell cycle analysis

SW620 and CTSC#18 cells were treated with azacitidine, romidepsin and IFN-α alone or in combination at the same dilutions used in drug cytotoxicity experiments for 48 h. After treatment, cells were harvested, washed with chilled PBS and permeabilized with cold 70% ethanol at 4°C, overnight. Then, cells were washed with cold PBS and resuspended in PBS containing Propidium Iodide (PI) (40 μg/ml) and RNase A (100 μg/ml). Flow cytometry was performed on Beckton–Dickinson Fluorescence Activated Cell Sorter (FACS, Franklin Lakes, NY) and samples analyzed employing the FlowJo Software.

### Cell death analysis

SW620 and CTSC#18 cells were treated with azacitidine, romidepsin and IFN-α, as described in the cell cycle assay. After treatment, cells were harvested and incubated with Annexin V-FITC and PI for the detection of cell death, according to the manufacturer's instructions. Cells were analyzed using FACS Calibur (Becton Dickinson). Data analysis was performed by WINMDI or Kaluza Software (Beckman Coulter). To determine the effects of the Caspase-3 inhibitor, cells were pretreated with z-DEVD-fmk (20 μM) for 2 h, stained and analyzed as above to detect cell death.

### Membrane CXCR4, CD133 and CRT expression

Immunofluorescence staining was used to detect CXCR4, CD133 and CRT exposure in SW620 and CTSC#18 cells treated with drugs. Briefly, cells were treated for 24 (CD133 in SW620 cells), 48 (CXCR4 in SW620 cells) and 72 h (CD133 and CXCR4 in CTSC#18 cells, CRT in both), as previously described and stained for 30 minutes at 37°C with mouse anti-CD133 (Miltenyi Biotec Inc, Germany), anti-CXCR4 and anti-CRT (Abcam) Abs. Samples were acquired by Gallios (Beckman Coulter) and analyzed in live cells (excluding 7AAD-positive dead cells) by Kaluza Software. Isotype-matched IgG Abs were used as control.

### DC generation

Peripheral blood mononuclear cells (PBMC) were obtained from the heparinized blood of healthy donors by Ficoll density gradient centrifugation (Seromed, Biochrom KG, Berlin). Monocytes were isolated from PBMC by column magnetic immunoselection (MACS CD14 microbeads, Miltenyi Biotec). Positively selected CD14^+^ monocytes were plated at the concentration of 2×10^6^ cells/ml in RPMI-1640 supplemented with 10% LPS-free fetal calf serum. Cultures were maintained for 3 days with 500 U/ml GM-CSF and 10000 IU/ml IFN-α. After 3 days of culture, non-adherent and loosely adherent cells namely IFN-α-DCs, were collected and used for experiments.

### Phagocytosis of apoptotic cells

SW620 and CTSC#18 cells were stained with PKH26 Fluorescent Cell Linker (Sigma-Aldrich), seeded and treated 24 h later with azacitidine, romidepsin and IFN-α alone or in combination. After 48 h treatment the floating cells were harvested, centrifuged and pellets were incubated for 4 h with IFN-α-DCs at 1:2 ratio. After co-culture, IFN-α-DCs were stained with anti-CD11c Ab (Becton Dickinson), and phagocytosis was detected by FACS analysis using FACS Calibur (Becton Dickinson) enumerating double-positive CD11c^+^/PKH26^+^ cells.

### Confocal microscopy

For CLSM analysis, SW620 and CTSC#18 cells were stained with PKH26 Fluorescent Cell Linker (Sigma-Aldrich), seeded and treated 24 h later with combined drugs for 48 h. Then, untreated and treated CRC cells were co-cultured with IFN-α-DCs for 4 h. Cells were fixed and images were observed through a 20X objective lens. Images were taken on an inverted microscope equipped with a confocal spectral imaging system (Olympus Fluoview 1000). Excitation light was obtained by a Diode Laser HeNe (561 nm) for PE. PE-Red emission was recorded from 583 to 628 nm. Images were analyzed by the C1-LCSI EZ-C1 Software.

### Animal studies

Female NOD-SCID mice purchased from Harlan Laboratories (Indianapolis, IN) were housed in the animal facility at the Istituto Superiore di Sanità (Rome, Italy) and manipulated in accordance with the local Ethical Committee guidelines. SW620 and CTSC#18 cells were injected subcutaneously (*s.c.*) 2×10^6^ and 1×10^6^, respectively. Mice were treated intraperitoneally (*i.p.*) with 0.25 mg/kg of azacitidine [64], 0.32 mg/kg of romidepsin [65], and 300000 IU/kg of IFN-α, dissolved in PBS, twice for week for three weeks. The control group of mice was injected with PBS.

### Statistical analyses

Each experiment has been repeated at least three times, yielding comparable results. Graphpad Prism v.5.03 (La Jolla, CA) and Microsoft Excel were used to graph data as mean ± S.D. or S.E.M. and to calculate p-values using Student's t-test. In all experiments, *p*-values ≤ 0.05 were considered as statistically significant.

## SUPPLEMENTARY FIGURES AND TABLE



## Abbreviations

A: azacitidine
Ab: antibody
AR: azacitidine and romidepsin combined treatment
ARI: azacitidine, romidepsin and IFN-α combined treatment
AU: arbitrary unit
CLSM: confocal laser scanning microscopy
CRC: colorectal cancer
CRT: Calreticulin
CSC: cancer stem cell
DC: dendritic cell
DNMTi: DNA methyltransferase inhibitor
FACS: Fluorescence Activated Cell Sorter
HDACi: histone deacetylase inhibitor
ICD: immunogenic cell death
IFN-α: IFN-α2b
I: IFN-α
IFN-α-DCs: IFN-α-conditioned DC
IFN-I: type I IFN
*i.p.*: intraperitoneal injection
MTS: 3-(4,5-dimethylthiazol-2-yl)-5-(3-carboxymethoxyphenyl)-2-(4-sulfophenyl)-2H-tetrazolium
NT: untreated cells
PBS: Phosphate Buffered Saline
PBMC: peripheral blood mononuclear cells
PI: Propidium Iodide
PI3K: phosphatidylinositol 3-kinase
qRT-PCR: quantitative reverse transcriptase PCR
R: romidepsin
RI: romidepsin and IFN-α combined treatment
*s.c.*: subcutaneous injection
SDS-PAGE: sodium dodecyl sulfate–polyacrylamide gel electrophoresis
