# Supplementary material for: IFN-α potentiates the direct and immune-mediated antitumor effects of epigenetic drugs on both metastatic and stem cells of colorectal cancer
Source: Oncotarget. 2016 Mar 25;7(18):26361–73. doi: 10.18632/oncotarget.8379 (PMC5041985; doi:10.18632/oncotarget.8379)
Supplement: Supplementary file 1 [file oncotarget-07-26361-s001.pdf]

IFN-α potentiates the direct and immune-mediated antitumor effects of epigenetic drugs on both metastatic and stem cells of colorectal cancer

SUPPLEMENTARY FIGURES AND TABLE

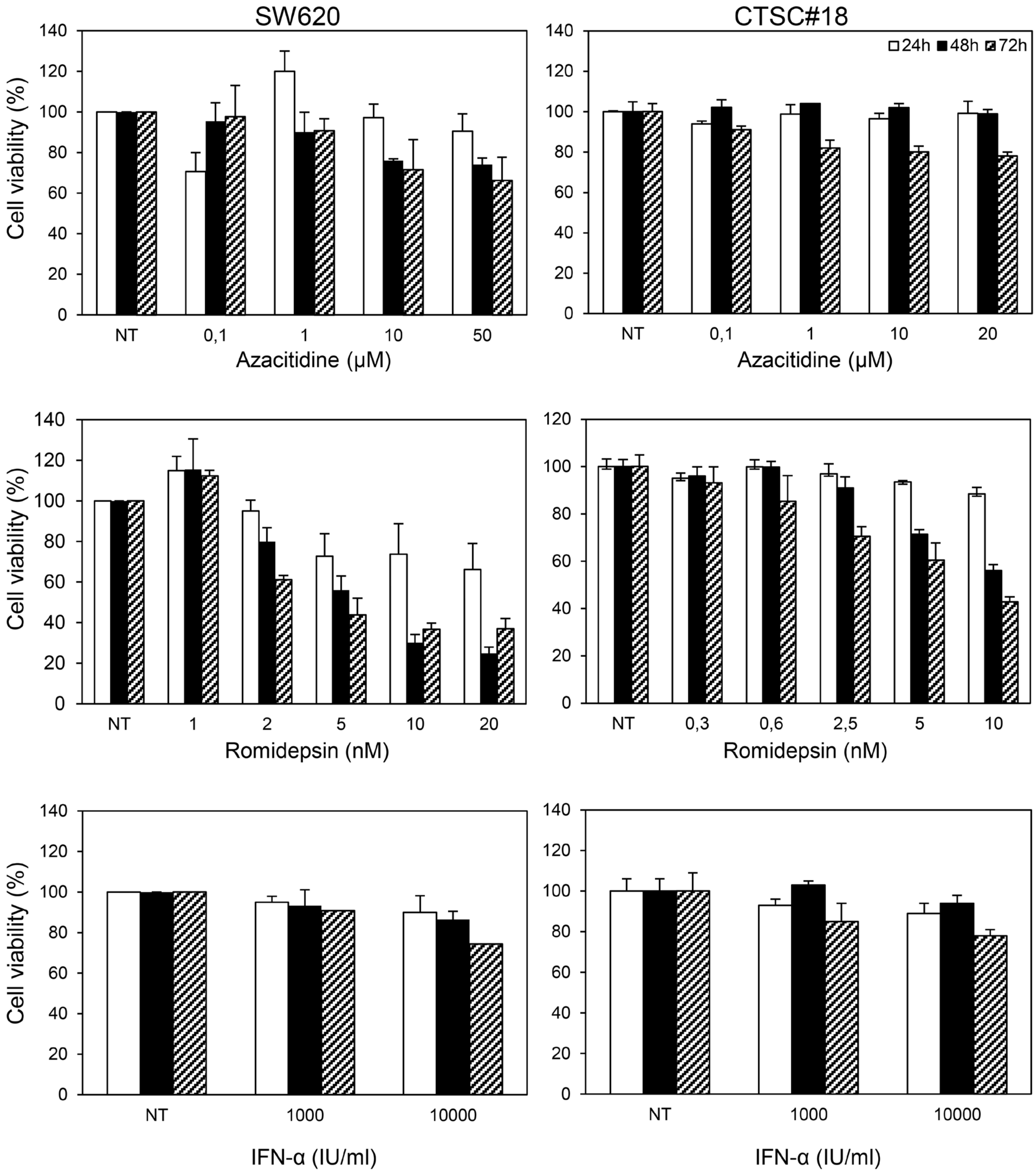

Supplementary Figure 1: Antiproliferative effects of azacitidine, romidepsin and IFN-α on metastatic cells and CSCs of CRC. Viability of drug-treated SW620 (left) and CTSC#18 (right) cells was evaluated at 48 and 72 h upon treatments by MTS assay. Drug-treated values are normalized to untreated cells (NT) at each time point. Experiments were performed in triplicate and each value represents mean ± S.D. of three independent experiments.

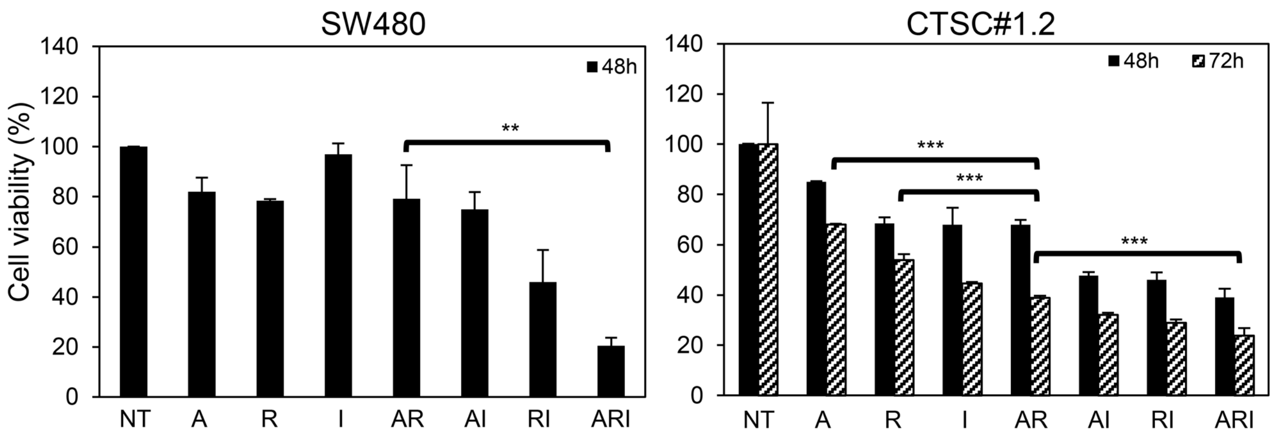

Supplementary Figure 2: Combined antiproliferative effects of azacitidine, romidepsin and IFN-α on SW480 and CSC line. Viability of SW480 and CTSC#1.2 cells was evaluated after 48 and 72 h drug treatments by MTS assay. Experiments were performed in triplicate and drug doses were used as in Supplementary Table 1. Each value is normalized to NT cells and data represent the means ± S.D. of three independent experiments. $^{**}P \leq 0.01$; $^{***}P \leq 0.001$.

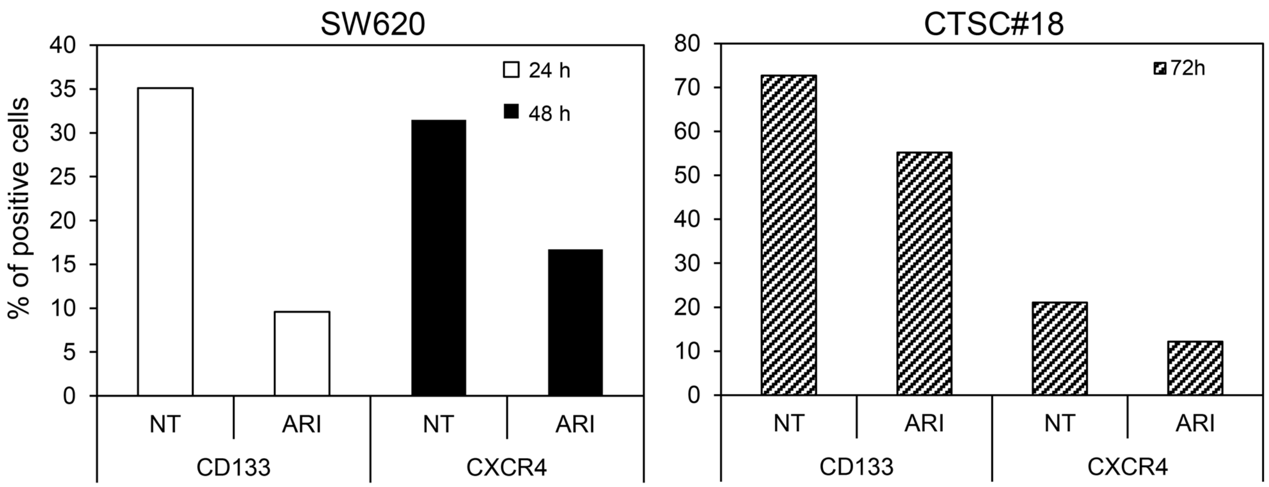

Supplementary Figure 3: ARI treatment down-modulates CD133 and CXCR4 surface expression in both metastatic cells and CSCs of CRC. FACS analysis of CD133 and CXCR4 in SW620 and CTSC#18 cells treated with ARI for 24 h (CD133 in SW620), 48 h (CXCR4 in SW620) and 72 h (CD133 and CXCR4 in CTSC#18).

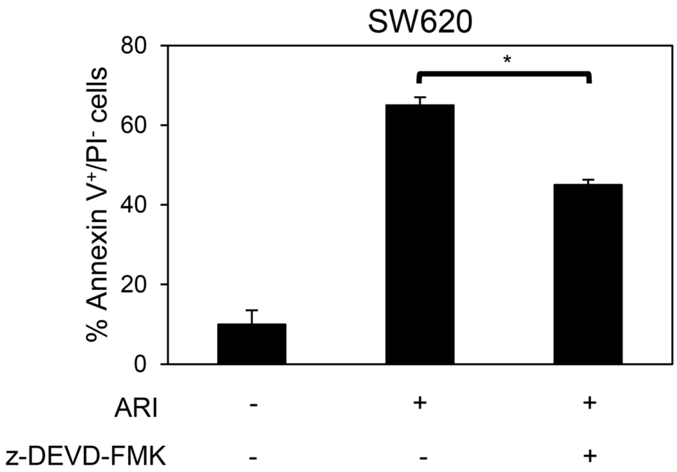

Supplementary Figure 4: Inhibition of Caspase-3 in ARI-treated SW620 metastatic cells. Cells were pretreated with z-DEVD-FMK for 2 h and stained to detect apoptosis by flow cytometry analysis. Early apoptotic (Annexin V+/PI−) and late apoptotic (Annexin V+/PI+) cells were considered for the analysis. Each value represents mean ± S.D. of three independent experiments. *$P \leq 0.05$.

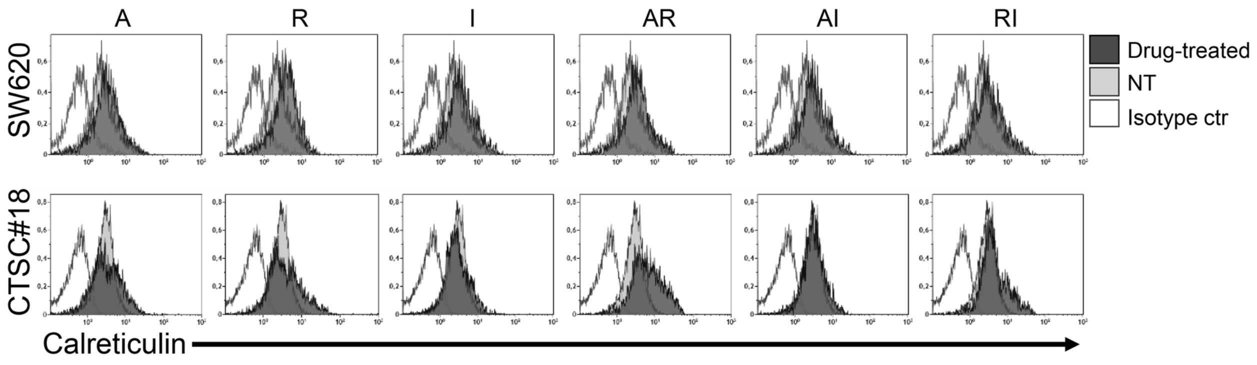

Supplementary Figure 5: CRT is not modulated by azacitidine, romidepsin and IFN-α as single agents and in double mixtures. SW620 and CTSC#18 cells were treated for 72 h with IFN-α and epigenetic drugs, alone or in combination, and CRT exposure on the cell membrane was assessed by flow cytometry analysis.

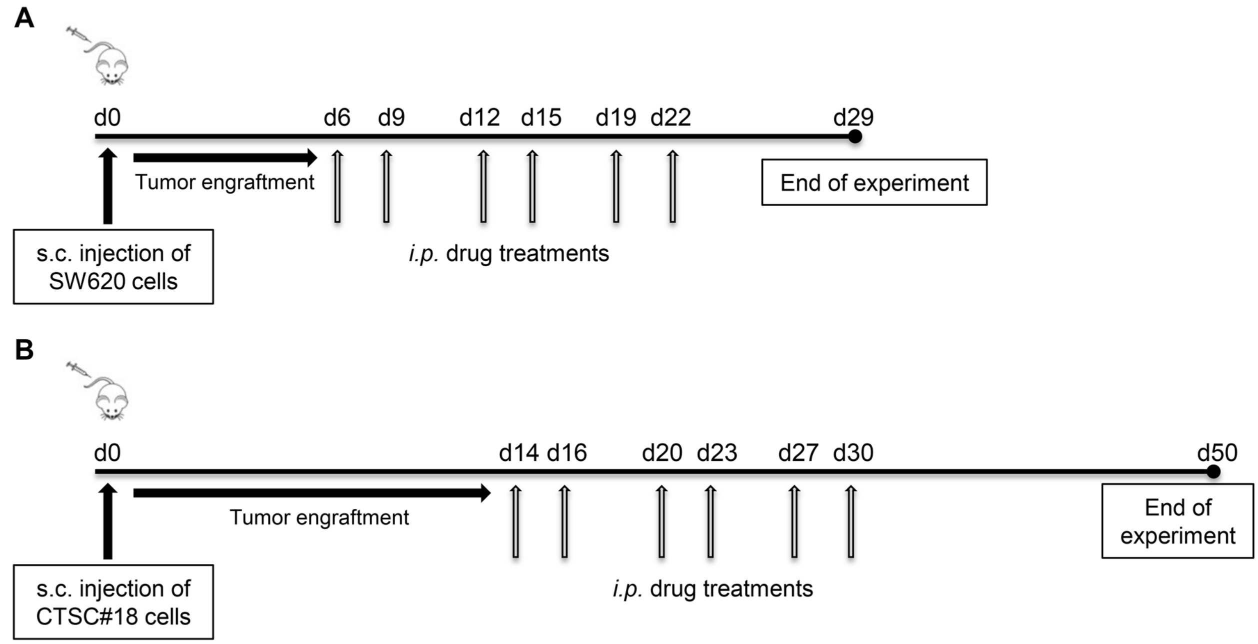

Supplementary Figure 6: Experimental set up and timeline of *in vivo* experiments performed with SW620 A. and CTSC#18 B. cells.

Supplementary Table S1: Potency of drugs in SW480 (48 h) and CTSC#1.2, CTSC#85, CTSC#CRO (all 72 h)

Treatments	SW480	CTSC#1.2	CTSC#85	CTSC#CRO
Azacitidine	IC20: 10 µM	IC30: 1 µM	IC18: 1 µM	IC30: 1 µM
Romidepsin	IC18: 2 nM	IC46: 0,6 nM	IC42: 0,6 nM	IC10: 0,6 nM
IFN-α	IC10: 10000 IU/ml	IC55: 10000 IU/ml	IC30: 10000 IU/ml	IC46: 10000 IU/ml

Data represent the mean of at least 3 independent experiments.
